# Shared Decision-Making for Drug-Drug Interactions: Formative Evaluation of an Anticoagulant Drug Interaction

**DOI:** 10.2196/40018

**Published:** 2022-10-19

**Authors:** Ainhoa Gomez Lumbreras, Thomas J Reese, Guilherme Del Fiol, Malinda S Tan, Jorie M Butler, Jason T Hurwitz, Mary Brown, Kensaku Kawamoto, Henrik Thiess, Maria Wright, Daniel C Malone

**Affiliations:** 1 Department of Pharmacotherapy Skaggs College of Pharmacy University of Utah Salt Lake City, UT United States; 2 Department of Biomedical Informatics Vanderbilt University Nashville, TN United States; 3 Department of Biomedical Informatics University of Utah Salt Lake City, UT United States; 4 Center for Health Outcomes and Pharmacoeconomic Research University of Arizona Tucson, AZ United States; 5 University of Arizona Tucson, AZ United States; 6 University of Heidelberg Heidelberg Germany

**Keywords:** decision making, shared, decision support systems, clinical, decision making, decision support, user-centered design, patient-centered care, risk management, drug interaction, pharmacotherapy, pharmacy, pharmaceutical, warfarin, unified theory of acceptance and use of technology, UTAUT, NSAID, anti-inflammatory, non-steroidal

## Abstract

**Background:**

Warnings about drug-drug interactions (DDIs) between warfarin and nonsteroidal anti-inflammatory drugs (NSAIDs) within electronic health records indicate potential harm but fail to account for contextual factors and preferences. We developed a tool called DDInteract to enhance and support shared decision-making (SDM) between patients and physicians when both warfarin and NSAIDs are used concurrently. DDInteract was designed to be integrated into electronic health records using interoperability standards.

**Objective:**

The purpose of this study was to conduct a formative evaluation of a DDInteract that incorporates patient and product contextual factors to estimate the risk of bleeding.

**Methods:**

A randomized formative evaluation was conducted to compare DDInteract to usual care (UC) using physician-patient dyads. Using case vignettes, physicians and patients on warfarin participated in simulated virtual clinical encounters where they discussed the use of taking ibuprofen and warfarin concurrently and determined an appropriate therapeutic plan based on the patient’s individualized risk. Dyads were randomized to either DDInteract or UC. Participants completed a postsession interview and survey of the SDM process. This included the 9-item Shared Decision-Making Questionnaire (SDM-Q-9), tool usability and workload National Aeronautics and Space Administration (NASA) Task Load Index, Unified Theory of Acceptance and Use of Technology (UTAUT), Perceived Behavioral Control (PBC) scale, System Usability Scale (SUS), and Decision Conflict Scale (DCS). They also were interviewed after the session to obtain perceptions on DDInteract and UC resources for DDIs.

**Results:**

Twelve dyad encounters were performed using virtual software. Most (n=11, 91.7%) patients were over 50 years of age, and 9 (75%) had been taking warfarin for more than 2 years (75%). Regarding scores on the SDM-Q-9, participants rated DDInteract higher than UC for questions pertaining to helping patients clarify the decision *(P=*.03), involving patients in the decision (*P=*.01), displaying treatment options *(P<*.001), identifying advantages and disadvantages (*P*=.01), and facilitating patient understanding (*P*=.01) and discussion of preferences (*P*=.01). Five of the 8 UTAUT constructs showed differences between the 2 groups, favoring DDInteract (*P*<.05). Usability ratings from the SUS were significantly higher (*P*<.05) for physicians using DDInteract compared to those in the UC group but showed no differences from the patient’s perspective. No differences in patient responses were observed between groups using the DCS. During the session debrief, physicians indicated little concern for the additional time or workload entailed by DDInteract use. Both clinicians and patients indicated that the tool was beneficial in simulated encounters to understand and mitigate the risk of harm from this DDI.

**Conclusions:**

Overall, DDInteract may improve encounters where there is a risk of bleeding due to a potential drug-drug interaction involving anticoagulants. Participants rated DDInteract as logical and useful for enhancing SDM. They reported that they would be willing to use the tool for an interaction involving warfarin and NSAIDs.

## Introduction

Approximately 8 million people in the United States received anticoagulants, including warfarin, in 2019 [1]. Warfarin is well known for having a multitude of drug-drug interactions (DDIs), with many avoided through health care provider awareness. However, studies found that up to 24% of people taking warfarin also received a prescription for nonsteroidal anti-inflammatory drugs (NSAIDs), and almost 50% of patients on warfarin have some form of ongoing pain [2,3]. Concomitant use of a NSAID and warfarin increases the risk of bleeding [4,5]. The risk of gastrointestinal (GI) bleeding is nearly 2-fold greater with this combination compared to using warfarin alone [5]. Furthermore, because ibuprofen and naproxen sodium are NSAIDs that are available over the counter (OTC), it is critical that patients understand the risks of taking the 2 medications concurrently. While the use of warfarin has declined since the approval of direct oral anticoagulants in 2013, it is the only anticoagulant indicated for mechanical valve replacement and is used by at least half of the patients taking oral anticoagulants [6-8]. Furthermore, patients’ knowledge of the adverse effects of NSAIDs is incomplete [9,10].

To improve health outcomes, stakeholders have advocated for the incorporation of shared decision-making (SDM) that encourages physicians to involve patients in selecting therapeutic treatments, among others [11]. SDM improves patient behavior and results in higher adherence to physician guidance, leading to improved health outcomes [12,13]. Currently, there are SDM tools available to facilitate decisions regarding nonvalvular atrial fibrillation anticoagulant treatment [14-18]. However, none of these SDM tools focus on or incorporate anticoagulant DDIs and their associated risks [19].

Current DDI alerting systems within electronic health records (EHRs) are clinician centric, suffer from high override rates, and do not account for patient and drug attributes that affect risk of harm from the interaction [20]. Furthermore, the primary source of DDI information (online DDI checkers) do not provide any personalized quantification of the risk of harm [21-24]. Accordingly, we developed DDInteract, a tool to facilitate SDM for patients receiving warfarin that calculates evidence-based risk of GI bleeding by considering an individual patient’s risk factors such as age, history of previous GI bleeding, and other medications [25]. We previously reported on the user-centered design and usability assessment of DDInteract. The purpose of this study was to conduct a formative evaluation comparing DDInteract to usual care (UC).

## Methods

### Study Design

This manuscript follows SUNDAE (Standards for Universal Reporting of Decision Aid Evaluations) guidelines [26]. We conducted a randomized formative evaluation of DDInteract compared to UC from March 2021 to August 2021. Formative evaluation is a research methodology used to rigorously examine factors that might influence the progress during the implementation of health care innovations [27]. We used a mixed methods approach, as we collected both quantitative and qualitative data.

### Ethics Approval

This project was approved by University of Utah Institutional Review Board (00127062). All participants signed the inform consent before participating in this study.

### Intervention

**Figure 1:**
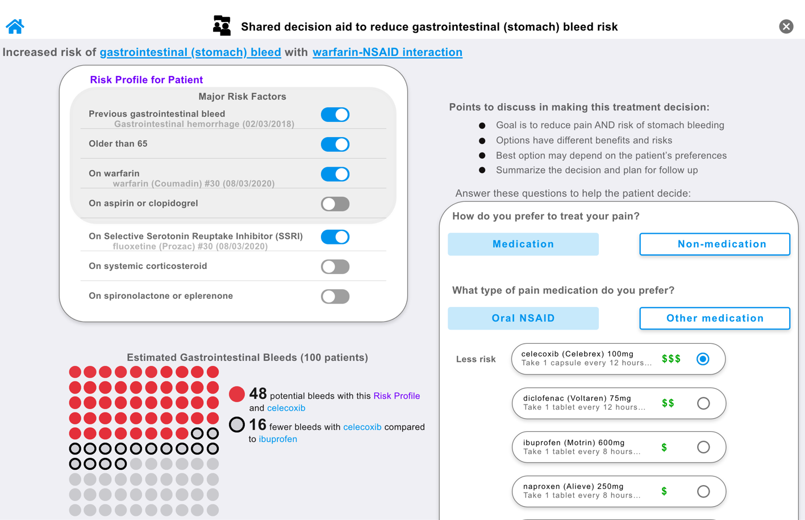
DDInteract tool (partial screen).

We designed DDInteract to be a free SDM tool that is integrated with the EHR to mitigate risk of harm due to DDIs involving warfarin and NSAIDs [25]. When launched from within an EHR, the tool retrieves risk factors from the patient’s record (eg, history of GI bleeding, age, medications) and calculates the patient’s risk of bleeding. A dynamic risk array displays and updates the risk of GI bleeding in real time based on clinician input, patient history, and medication choices (Figure 1). When an NSAID is selected, the risk array changes to reflect the increase in risk due to the combined effects of both agents on risk of bleeding. The tool also (1) provides information on nonmedication treatments for pain, such as acupuncture and physical therapy; (2) includes strategies to reduce risk of GI bleeding through use of proton pump inhibitors; (3) facilitates the medication prescription process; (4) automates clinical documentation of the SDM discussion; and (5) provides patient education in an “after visit summary.” Further details about DDInteract functionality are described elsewhere, and the tool is available for demonstration purposes on the DDInteract website [28].

### Study Settings

We randomized physician-patient dyads to participate in simulated clinical encounters using either DDInteract or conventional drug information resources (UC) to treat a patient on warfarin who is also seeking relief of pain by using an NSAID. Physicians in both groups (DDInteract or UC) received access to various online DDI resources (eg, Micromedex), while those in the intervention group also had access to DDInteract.

### Recruitment: Inclusion and Exclusion Criteria

We recruited physicians through electronic communications, presentations to medical groups, and snowball sampling. The inclusion criteria for physicians required experience with treating patients on oral anticoagulants. We recruited patients from the University of Utah Health System, including the Division of Cardiology and the Anticoagulation Service. Patient participants needed to be older than 21 years of age, fluent in English, and currently taking an oral anticoagulant.

### Encounters

We integrated and deployed DDInteract with the Logica sandbox EHR environment using the Substitutable Medical Applications and Reusable Technologies (SMART) guidelines on Fast Health Interoperability Resources (FHIR) standards [29]. Details on the technical architecture including interoperability approach are available elsewhere [30]. Physician-patient dyads participated in a simulated virtual encounter using an online meeting software and provided consent prior to starting the simulated encounter. We instructed both physicians and patients to conduct themselves as they would in an encounter where an anticoagulated patient wants to use ibuprofen to treat their pain but has concerns about bleeding. Participants permitted us to record the encounters. Physicians and patients received instructions separately via breakout rooms and asked study investigators any questions prior to the simulated visit.

Physicians randomized to the DDInteract group received a link to a video demonstrating the functionality of DDInteract, while those randomized to the UC group received information on the main topic and of online drug information sources that can screen for drug interactions (see Table S1 in the Multimedia Appendix 1). Physicians in both groups received access to online resources for DDIs to use during the encounter at their discretion. After the simulated encounters, both physicians and patients participated in a semistructured interview with a member of the research team to discuss the encounter and use of DDInteract and online drug information resources. Upon concluding the simulated encounter, participants completed an online survey containing the instruments described in the sequent section.

### Outcomes

The primary outcome was quality of SDM according to the 9-item Shared Decision-Making Questionnaire (SDM-Q-9) questionnaire assessed by physicians. The physician postsimulation survey included items in 6 groupings: (1) SDM-Q-9 scale [31]; (2) adapted System Usability Scale (SUS) [32]; (3) National Aeronautics and Space Administration (NASA) Task Load Index (NASA-TLX) [33]; (4) Unified Theory of Acceptance and Use of Technology (UTAUT) [34], including the performance expectancy, effort expectancy, attitude toward using technology, social influence, facilitating conditions, self-efficacy, anxiety, and behavior constructs; (5) Perceived Behavioral Control (PBC) scale [35]; and (6) questions pertaining to participants’ demographics and professional experience. The patients’ survey included 6 items adapted from the SUS scale, 11 items adapted from the Decisional Conflict Scale (DCS) [36], and questions about their demographics and anticoagulation treatment.

### Statistical Analysis

We calculated the mean and standard deviation for each item/construct from the various scales and instruments. For SDM quality, which was the primary outcome, we conducted a 2-group Student *t*-test assessing differences for DDInteract versus UC as rated by physicians. A similar approach was used to test for differences in secondary measures including the SUS, NASA-TLX, and UTAUT. The PBC scale rated by physicians was also analyzed using the same approach, as well as items on the DCS. The total time of the simulated encounter for both groups was compared using Student *t*-test. All statistical comparisons used a 2-tailed test with an alpha <.05. Given the relatively small sample size, no adjustments were made for multiple comparisons. Since we could not assume that the populations were normally distributed, we estimated the Mann-Whitney U test for all the outcomes results reported. We also reviewed transcriptions of the post encounter semistructured interviews and highlighted the most insightful comments. All transcriptions were reviewed, and the principal investigators extracted the most relevant and repeated comments. Data were analyzed using Microsoft Excel for Mac 2016 (IBM Corp) and Stata (version 17, basic edition).

## Results

### DDInteract Tool and UC Survey Results

A total of 12 physician-patient dyads completed the formative evaluation. We randomized 6 dyads to DDInteract and 6 to UC. Participant demographics appear in Table 1. The UC group had 1 more female physician and 2 more female patients than the DDInteract group. All patients and 9 (75%) of the physicians reported being Caucasian. There was no difference in physicians’ self-reported experience managing patients with warfarin between the 2 groups (DDInteract 68.8, SD 31.5 vs UC 77.7, SD 20.6, from a 0-100 scale, *P*=.58).

The primary outcome of interest (SDM), as measured by the SDM-Q-9, appears in Table 2. Perceptions of SDM differed significantly between DDInteract and UC groups for 6 of the 9 attributes (*P*<.05) and include: facilitated the discussion on preferences, assisted patient comprehension, presented different options, presented advantages and disadvantages of the different treatment options, involved the patient, and provided clarity on the decision to be made. Not surprisingly, the largest difference between the groups was with respect to presentation of different treatment options (mean 5.7, SD 0.5 vs mean 1.8, SD 1.2, *P*<.001) and presentation of advantages and disadvantages (mean 5.0, SD 1.1 vs mean 2.8, SD 1.2, *P*=.008) for DDI and UC, respectively.

The task load assessed by the NASA-TLX index, scaled from zero (low/easy/successful) to 10 (high/demanding/unsuccessful), was lower for the DDInteract group than the UC group; it was not statistically significant but trended toward significance (mean 2.0, SD 1.2 vs mean 4.2, SD 2.1, *P*=.08, respectively) (Table 3). Participants indicated that DDInteract took significantly less effort to use than the tools in UC (mean 0.8, SD 0.7 vs mean 7.3, SD 3.7, *P*=.008, respectively), but none of the other constructs differed significantly between the study groups.

Patient-reported scores on the adapted SUS scale did not differ between groups, but physician ratings differed significantly, with DDInteract perceived as more logical, efficient, and helpful/effective than UC and SDM perceived as more valuable than using traditional DDI tools in UC (*P*<.05) (Table 4).

Physicians’ responses on a Likert scale ranging from 1 (strongly disagree) to 7 (strongly agree) in the DDInteract group differed from those in the UC group on the following UTAUT constructs: performance expectancy (mean 5.6, SD 1.2 vs mean 3.4, SD 1.7, *P*<.001), effort expectancy (mean 6.8, SD 0.5 vs mean 5.2, SD 1.6, *P*<.001), attitude toward using technology (mean 5.7, SD 1.1 vs mean 3.9, SD 1.8, *P*<.001), social influence (mean 5.5, SD 0.8 vs mean 4.1, SD 1.8, *P*<.001), and anxiety (mean 1.4, SD 0.5 vs mean 2.5, SD 1.5, *P*<.001), respectively (Table 5). In general, it appears that physicians perceived DDInteract to perform better, require less effort, improve attitudes toward technology, be supported by administration and colleagues, and reduce perceived anxiety more compared to UC.

Table 6 shows the physicians’ assessment of PBC. Physicians exposed to DDInteract were more likely to indicate using SDM in the clinic compared to physicians randomized to UC (mean 6.8, SD 0.4 vs mean 6.0, SD 0.6, *P*=.02, respectively). DDInteract physicians were also more likely to perceive conducting SDM without extending the duration of the visit than UC physicians (mean 5.5, SD 0.5 vs mean 3.7, SD 1.5, *P*=.03). However, the mean duration of the encounters increased 5 minutes on average for the DDInteract, but this increase did not significantly differ between the DDInteract and UC groups (mean 17.6, SD 5.4 minutes vs mean 12.7, SD 4.8 minutes, *P*=.13). No other PBC items differed significantly between the 2 groups. There were no differences between the groups in patients’ ratings on the DCS (Table S2 in Multimedia Appendix 1).

The results of the nonparametric tests for the different outcomes (SDM-Q-9, UTAUT, SUS, NASA-TLX, PBC, and DCS) did not differ in terms of statistical significance from the ones presented above (Tables S3-S9 in Multimedia Appendix 1). Qualitative narrative data were collected to gain insight into participants thoughts on SDM and DDI.

**Table 1:** Participants’ sociodemographic, professional, and treatment characteristics.

Characteristics			Physicians								Patients					
			DDInteract (N=6), n (%)		UC^a^ (N=6), n (%)		*P* value			DDInteract (N=6), n (%)			UC (N=6), n (%)		*P* value	
**Age**								.34								.39
	21-29	1 (16.7)		—					—			—				
	30-39	2 (33.3)		1 (16.7)					—			—				
	40-49	3 (50)		2 (33.3)					1 (16.7)			—				
	50-59	—		1(16.7)					3 (50)			2 (33.3)				
	≥60	—		2 (33.3)					2 (33.3)			4 (66.7)				
Female sex			2 (33.3)		3 (50)		.56			3 (50)			5 (83.3)		.22	
**Race**								.37								>.99
	American Indian/ Alaska Native	—		—					—			—				
	Asian/Pacific Islander	—		1 (16.7)					—			—				
	African American	—		—					—			—				
	Hispanic	1 (16.7)		—					—			—				
	White/ Caucasian	4 (66.7)		5 (83.3)					6 (100)			6 (100)				
	Multiple ethnicity/other	1 (16.7)		—					—			—				
**Clinical experience**								.64								—
	<5 years	1 (16.7)		1 (16.7)					—			—				
	6-10 years	2 (33.3)		1 (16.7)					—			—				
	11-15 years	2 (33.3)		1 (16.7)					—			—				
	≥16 years	1 (16.7)		3 (50)					—			—				
**Level of education**								—								.99
	College graduate	—		—					2 (33.3)			3 (50)				
	Attended college	—		—					1 (16.7)			1 (16.7)				
	High school graduate	—		—					1 (16.7)			1 (16.7)				
	Completed graduate school	—		—					2 (33.3)			1 (16.7)				
**Time on anticoagulant**								—								.50
	≤6 months	—		—					2 (33.3)			1 (16.7)				
	>6 months ≤2 years	—		—					0			0				
	>2 years	—		—					4 (66.7)			5				
**Time in clinical care**								.08								—
	≤20% week	—		2 (33.3)					—			—				
	21%-40% week	1 (16.7)		1 (16.7)					—			—				
	41%-60% week	—		2 (33.3)					—			—				
	61%-80% week	—		—					—			—				
	≥81% week	5 (83.3)		1 (16.7)					—			—				
**Specialty**								.56								—
	Internal medicine	3 (50)		3 (50)					—			—				
	Family medicine	1 (16.7)		2 (33.3)					—			—				
	Geriatric medicine	1 (16.7)							—			—				
	Other	1 (16.7)		1 (16.7)					—			—				

^a^UC: usual care.

**Table 2:** The 9-item Shared Decision-Making Questionnaire (SDM-Q-9).

	Physicians, mean (SD)		*P* value
	DDInteract	UC^a^	
Clarity of the SDM^b^	4.5 (0.8)	3.3 (0.8)	.03
Involve the patient	5.5 (0.5)	3.2 (1.3)	.01
Different option presented	5.7 (0.5)	1.8 (1.2)	<.001
Presented advantages/disadvantages	5.0 (1.1)	2.8 (1.2)	.01
Assisted patient comprehension	4.8 (0.4)	2.7 (1.4)	.01
Facilitated discussion on preferences	5.2 (0.8)	3.0 (1.3)	.01
Evaluated options	5.0 (0.0)	4.7 (0.8)	.36
Co-selected a treatment option	5.2 (0.4)	5.0 (0.9)	.69
Reach an agreement	5.7 (0.5)	5.3 (0.8)	.42

^a^UC: usual care.

^b^SDM: shared decision-making.

**Table 3:** Adapted National Aeronautics and Space Administration Task Load Index (NASA-TLX)^a^.

NASA^b^ Task Load Index construct	Questions	Physicians, mean (SD)			*P* value
		DDInteract	UC^c^		
Mental demand	How much mental effort was required to decide on the patient’s treatment?	3.8 (1.0)	4.7 (3.2)	.57	
Physical effort	Was using the DDInteract decision tool easy or demanding?	0.8 (0.7)	7.3 (3.7)	.008	
Temporal demand	How much time did it take to investigate the drug interaction during the simulated visit?	1.8 (1.6)	3.6 (3.5)	.28	
Effort	How hard did you have to work to make a decision with the patient?	2.3 (1.0)	3.6 (2.6)	.30	
Performance	How successful do you think you were in making a shared decision with the DDInteract decision tool?	1 (0.9)	1.7 (1.8)	.45	
Total average		2.0 (1.2)	4.2 (2.1)	.08	

^a^Likert scale from 0 (low/easy/successful) to 10 (high, demanding, unsuccessful).

^b^NASA: National Aeronautics and Space Administration.

^c^UC: usual care.

**Table 4:** Modified System Usability Scale (SUS).

Items	Patients, mean (SD)			*P* value		Physicians, mean (SD)			*P* value
	DDInteract	UC^a^			DDInteract		UC		
It was logical	4.7 (0.5)	4.8 (0.4)	.65		4.8 (0.4)		3.7 (1)	.03	
It was efficient	4.5 (0.5)	4.4 (0.5)	.77		4.7 (0.5)		3.5 (1)	.04	
Helpful/effective in the decision-making process	4.7 (0.5)	4.4 (0.9)	.57		4.5 (0.5)		2.8 (1.2)	.02	
The SDM^b^ using the tool was valuable	4.5 (0.5)	4.8 (0.4)	.26		4.7 (0.5)		2.8 (0.7)	<.001	
The tool was valuable	N/A^c^	N/A	N/A		4.3 (0.5)		2.8 (1.2)	.02	
Easy to use	4.5 (0.5)	4.4 (0.9)	.83		4.8 (0.4)		3.2 (1.5)	.04	
Enjoyed the experience	4.7 (0.5)	4.8 (0.4)	.55		N/A		N/A	N/A	
Learned something from this experience	4.8 (0.4)	4. 7 (0.8)	.67		4.5 (0.5)		2.8 (1.7)	.06	

^a^UC: usual care.

^b^SDM: shared decision-making.

^c^N/A: not applicable.

**Table 5:** Unified Theory of Acceptance and Use of Technology (UTAUT) responses.

	Physicians, mean (SD)		*P* value
	DDInteract	UC^a^	
Performance expectancy	5.6 (1.2)	3.4 (1.8)	<.001
Effort expectancy	6.8 (0.5)	5.2 (1.6)	<.001
Attitude toward using technology	5.7 (1.1)	3.9 (1.8)	<.001
Social influence	5.5 (0.9)	4.1 (1.8)	<.001
Self-efficacy	5.3 (2.1)	4.6 (2.2)	.26
Facilitating conditions	5.1 (1.9)	5.4 (1.7)	.58
Anxiety	1.4 (0.5)	2.5 (1.5)	<.001
Behavioral	5.5 (1.0)	4.7 (2.9)	.53

^a^UC: usual care.

**Table 6:** Perceived Behavioral Control (PBC) scale.

Item	Physicians, mean (SD)		*P* value
	DDInteract	UC^a^	
I am convinced that I can share decision‐making in the clinic	6.8 (0.4)	6 (0.6)	.02
I have control over the level of SDM^b^ that is accomplished in the clinic	6.2 (0.8)	5.7 (0.5)	.21
I can perform SDM without extending the duration of the consultation	5.5 (0.5)	3.7 (1.5)	.03
Knowledge about SDM is important in order to apply SDM	6.5 (0.5)	5.7 (1.4)	.20
Communication skills are important for SDM	7 (0)	6.7 (0.5)	.17
Patients are motivated to participate in SDM	6 (0.9)	5.5 (1.2)	.44
In general patients have enough knowledge, intelligence and understanding needed for SDM	6 (0.9)	5 (1.7)	.23

^a^UC: usual care.

^b^SDM: shared decision-making.

### Physician and Patient Perceptions Regarding the DDInteract Tool and Online DDI Resources

After the virtual encounters, both clinicians and patients discussed the simulation using a semistructured interview format to elicit their impressions of DDInteract and online DDI resources. Below are select quotes from the participants. In general, DDInteract was well received by the physicians, as indicated by the following statements:

I like it; it helps patients visualize the risk instead of me just talking statistics, they see the risk in the more obvious way. I like the intervention that shows the risk of bleeding, you can increase more risk factors for bleeding, the idea is excellent.

Physician #4, male

I really like it. Whenever I can, I like to show something visual while taking to the patient. It is very user friendly, simple. It is not overly complicated.

Physician #8, female

Concerns about extra time involved in using DDInteract were not evident among the participants. One physician stated:

I am trying to imagine having this conversation without the tool. I don't even think the tool would even make it longer; I think what it does is cut down on having to overly explain things, cut down on the feeling that I have to reemphasize things because it was a visual tool, and the patient is seeing what I am seeing, so they don't ask for repeating. So I probably save some questions too. In all I think it would save some time.

Physician #8, female

Another physician felt that the tool would encourage more conversations with the patient and help them understand the risks associated with bleeding and anticoagulation treatment:

I think it was fast to use. I see thrombosis patients, so I do a lot of counseling about warfarin or anticoagulation. I think I tend to assume that people have already been through education, but this would be nice because it will slow me down and help me actually understand what the risk is, which I might just summarize very quickly, but I think this would be really helpful from a patient standpoint to actually, like, seeing is something that explains it a little better.

Physician #10, female

When patients were asked about the icon array in DDInteract as an approach to display the risk of bleeding, some comments included:

I think seeing the graphic portrayal of the different risk levels and how to treat the pain was very helpful. I think it was well done…I thought this tool was much more thorough than the information that I've gotten in the normal clinic visit. I did not know the number of bleeds per 100 patients; that has never been discussed with me during my doctor visit.

Patient #10, female

The icon array definitely makes sense to me.

Patient #12, male

I learn better by what I see.

Patient #9, female

I think the chart with different numbers showing different reactions you get by taking ibuprofen kind of spells it out like black and white for me.

Patient #8, male

Table S10 in Multimedia Appendix 1 provides additional comments made by the physicians and patients.

## Discussion

### Principal Results

Results of the formative evaluation suggest that there was a positive opinion of DDInteract as a potentially useful tool to help facilitate SDM concerning concomitant therapies that could result in a DDI. Physicians perceived the tool as intuitive, easy to use, and not increasing the amount of time during an encounter. After the simulated encounter, patients exposed to the DDInteract tool commented that they liked the way the information was presented, including the quantitative estimation of the risk and how risk changed according to patient factors in the user interface.

Physicians that used the DDInteract reported enhanced SDM compared to physicians randomized to UC using a standardized and validated tool (SDM-Q-9) [31,37]. This study demonstrates that DDInteract assisted in facilitating discussion, increasing patient comprehension, providing different treatment options with their respective advantages and disadvantages, and providing clarity around the treatment choices and the final decision. This contrasts with current warnings about potential interactions that are clinician orientated and not designed to be shared with patients.

Due to the ubiquitous use of NSAID pain relievers, including both prescription and over-the-counter products, patients play an important role in preventing harm from DDIs. Through education, patients can reduce harm by avoiding interacting OTC medications and monitoring signs and symptoms of adverse consequences [38]. One of the most important aspects of DDInteract is the risk array, which dynamically changes when risk factors or medications are selected. Usability studies have demonstrated that this approach is appealing and helpful for displaying the risk of harm [25]. Furthermore, physicians using DDInteract during the encounter received no additional training in SDM but rated SDM aspects higher than physicians randomized to UC.

### Secondary Results

Results from the SUS index suggest that physicians rated DDInteract as more logical, efficient, valuable, easy to use, and helpful/effective than physicians in the UC who rated online compendia and traditional EHR tools. This finding is not surprising given the lack of patient-specific information provided by both EHR systems and online resources for DDIs. Only 1 attribute of the SUS (“learned something”) did not differ significantly between groups (*P*=.06). Patient perceptions of DDInteract and UC tools did not differ on the SUS scale, a finding that might be attributable to patients wanting more information about DDIs [39,40].

DDInteract appeared to be intuitive and easy to use according to participant ratings on the NASA-TLX and UTAUT instruments. These results support those of a previously reported usability study that was conducted when designing the tool [25]. The NASA-TLX has been previously used to evaluate the workload associated with the new Clinical Decision Support Tool similar to DDInteract [41]. Compared to UC, physicians rated DDInteract higher on the UTAUT domains of performance expectancy, effort expectancy, attitude toward the technology, and social influence. Furthermore, DDInteract was associated with lower anxiety than UC resources for DDIs. These findings align with a metaregression of studies that evaluated effective clinical decision support using the UTAUT model and suggested that effort expectancy, facilitating conditions, and performance expectancy had a significant impact on clinician behavior [42]. Numerous studies evaluated medication-related CDS using the UTAUT, including studies examining a comprehensive CDS suite of tools called Sentri7/Quantifi [43], use of a decision aid for psychotic disorders [44], medication management in oncology settings [45], and a tool to help remind HIV positive men to use antiviral therapies when undertaking sexual activities [46]. However, to our knowledge, none have applied the UTAUT model to DDI-specific SDM.

Patient responses to the DCS did not differ between groups, which is unsurprising. Studies using the DCS in the context of nonvalvular atrial fibrillation (NVAF) found mixed results with patient decision aids [47,48]. NVAF decision aids focused on anticoagulant treatments show lower DCS scores or a decrease in the DCS score after using the decision aid, but these differences were not significantly different when compared with groups not using such aids [18,47,48]. In addition, the small sample size may contribute to the lack of significant differences between the groups with respect to the DCS in our study.

The time required to use CDS and SDM applications is a common concern among clinicians. Our study found that physicians did not perceive DDInteract to affect the amount of time or effort spent with patients (PBC scale question on performing SDM without extending the time of consultation favored DDInteract, *P*=.03), though there was a nonsignificant 5-minute difference in the time to conduct the simulated encounter, with DDInteract encounters being longer. During the debrief, 1 physician proposed that DDInteract could actually save time because it visually explains the risk of harm and various treatment options, as compared to having to explain the risks verbally. This disconnect between observed time to use the DDInteract and perceived time to use the tool is probably due to clinicians, who had no previous experience with the tool, having to learn how to use DDInteract during the simulation. Clinicians found the tool easy to use and intuitive, indicating a reduced time to use the tool in future encounters.

DDInteract is a novel approach to providing information about potential interactions. Currently, online drug compendia and warnings within EHR systems have numerous issues [49]. They do not account for individual patient risk factors or provide quantitative estimates of the risk. Additionally, they lack the ability to explore changes in the risk with therapeutic alternatives and are almost completely text based without any visual display of information. Furthermore, they may contain incorrect information. For example, Drugs.com currently states that the mechanism to control bleeding risk in patients on warfarin and ibuprofen is to closely monitor the international normalized ratio (INR); however, concurrent use of NSAIDs and warfarin interaction is a pharmacodynamic interaction that will not affect the INR [24]. Studies have found patients’ INRs to be within range among individuals experiencing bleeding and taking both medications, and a systematic review did not find an effect on INR for ibuprofen or naproxen [50,51]. A review of 7 Chinese apps that support DDI checking and target health care professionals had tremendous variation in information about DDIs, with over 50% lacking accurate information on DDIs [52]. Although DDInteract is currently limited in scope in terms of the interactions it includes, the approach is generalizable to other interactions, and efforts are underway to expand these interactions.

### Limitations

This study has several limitations that should be considered when interpreting the results. We recruited fewer participants than our a priori sample size (16 dyads), but we still observed significant differences between the study groups in terms of the primary outcome of SDM quality and several secondary outcomes of interest. COVID-19 protocols and restrictions limited our recruitment of subjects because clinic visits have been in part shifted to telehealth, making it more difficult to contact patients, and because physicians were overburdened with demands due to the pandemic [53,54]. In addition, this study focused on a specific DDI involving warfarin, which is being prescribed less because of alternative anticoagulants that require less frequent monitoring. Several physicians commented that they would have liked to see DDInteract incorporate other anticoagulants and other bleeding risks beyond GI. As stated previously, efforts are underway to broaden the interactions incorporated into the tool. Another limitation is that the clinical scenario required dyads to role-play a scenario that may not occur in clinical practice, where a patient is seeking advice from the physician about treating pain while on an anticoagulant. While this scenario may be infrequent in most practice settings, previous studies have found this to occur in up to 25% of patients receiving warfarin [2,3]. Furthermore, many physicians in the study indicated they were aware of the interaction when researchers explained the scenario prior to the simulated visit. Thus, many practitioners may have behaved differently when provided with DDInteract or other DDI information. Nonetheless, after the simulated visit, many practitioners exposed to DDInteract were enthusiastic about the tool. Therefore, we believe DDInteract may be useful in many practice settings, especially as the tool is expanded to support SDM for other drug interactions, such as other oral anticoagulants.

### Conclusions

DDInteract is a novel tool designed to facilitate SDM during encounters concerning potentially harmful DDIs. The tool improved SDM, was not overly burdensome according to the NASA-TLX and UTAUT scales, and was generally well received by both physicians and patients. However, further research is needed to evaluate the impact of DDInteract on exposure to harmful DDIs and other important health outcomes.

## Appendix

Multimedia Appendix 1 Supplementary Tables S1-S10.

## Abbreviations

DCS: Decisional Conflict Scale
DDI: drug-drug interaction
EHR: electronic health record
FHIR: Fast Health Interoperability Resources
GI: gastrointestinal
INR: international normalized ratio
NASA-TLX: National Aeronautics and Space Administration Task Load Index
NSAID: nonsteroidal anti-inflammatory drug
NVAF: nonvalvular atrial fibrillation
OTC: over the counter
PBC: Perceived Behavioral Control
SDM: shared decision-making
SDM-Q-9: 9-item Shared Decision-Making Questionnaire
SMART: Substitutable Medical Applications and Reusable Technologies
SUNDAE: Standards for Universal Reporting of Decision Aid Evaluations
SUS: System Usability Scale
UC: usual care
UTAUT: Unified Theory of Acceptance and Use of Technology
