# Supplementary material for: Shared Decision-Making for Drug-Drug Interactions: Formative Evaluation of an Anticoagulant Drug Interaction
Source: JMIR Form Res. 2022 Oct 19;6(10):e40018. doi: 10.2196/40018 (PMC9631167; doi:10.2196/40018)
Supplement: Multimedia Appendix 1 [file formative_v6i10e40018_app1.docx]

Supplemental Material Formative Evaluation DDInteract

Table 1s Usual Care physician’s information

Please lead this encounter as your daily practice, remember we won’t be evaluating your practice. We want you to focus on a known drug-drug interaction (DDI) and the information you need to convince the patient to avoid the combination, showing him/her the risk of the harm when the on the DDI. Consider the SDM as “*an approach where clinicians and patients share the best available evidence when making decisions, and where patients are supported to consider options, to achieve informed preferences*”

| **Patients asking about a drug-drug interaction (DDI)** |
| --- |
| 1. Engage the patient by introducing them to the potential issue with NSAIDs and warfarin (e.g., Increased risk of gastrointestinal bleed with warfarin-NSAID interaction). 2. Make a flexible recommendation based on patient factors and your knowledge of the DDI. You could show some information regarding DDI and their potential risk (gastrointestinal bleeding for this encounter) from the following Drug Interactions Checkers:  - [WebMD](https://www.webmd.com/interaction-checker/default.htm) - [Drugs.com](https://www.drugs.com/drug_interactions.html) - [Medscape](https://reference.medscape.com/drug-interactionchecker) - [Epocatres](https://reference.medscape.com/drug-interactionchecker) (you have to sign in) - [UptoDate](https://www.uptodate.com/drug-interactions/?source=responsive_home#di-druglist) (log in to CIS)   We would recommend you to share your screen with the patient to show him/her any information which would help the patient to understand the risk.  In order to inform the patient on your prescription and be able to provide the patient some information material.   1. Consider showing the patient any information material or webpage you think could be convenient to discuss benefits and harms. |
| 1. Select final option and discuss the decision |

Table 2s: Patients Decisional Conflict Scale (DCS) survey results.

|  | **Patients**  **(mean, SD)** | |  |
| --- | --- | --- | --- |
|  | **DDInteract**  **N=6** | **UC**  **N=6** | ***p-value*** |
| Decision is hard for me to make | 3.3 (1.2) | 3.5 (1.4) | 0.83 |
| Unsure what to do in this decision | 3.7 (1.0) | 3. 7 (0.8) | 1.00 |
| Clear what choice is best for me | 1.8 (0.75) | 2.2 (1.2) | 0.57 |
| I am aware of the choices I have to protect myself from drug interactions | 1.8 (1.7) | 1. 7 (0.5) | 0.76 |
| I know the benefits of avoiding drug interactions | 1. 7 (1.2) | 1. 7 (0.8) | 1 |
| I know the potential for harm for drug interactions | 1. 7 (0.8) | 1.5 (0.5) | 0.69 |
| I need more advice and information about my choices | 3 (1.3) | 2. 7 (1.2) | 0.65 |
| It’s hard for me to decide if the benefits are more important to me than the risks, or opposite | 3 (1.3) | 3 (1.1) | 1 |
| I feel I have made an informed choice | 1.7 (0.8) | 1.8 (0.7) | 0.72 |
| My decisions show what is most important for me | 1.8 (0.9) | 1.7 (0.5) | 0.72 |
| I expect to stick with my decision | 1.8 (0.9) | 2.2 (0.7) | 0.53 |

Likert scale (1 Strongly Agree to 5 Strongly Disagree)

Patient’s rating of the 11 items of the DCS was not different between the groups, with agreement scores for “being a hard decision to make” and “unsure what to do in this decision” and disagreement for “knowing the potential for harm for drug interactions”.

**NON-PARAMETRIC RESULTS**

This were conducted with Stata

Table 3s.SDM-9

| **SDM-9 item** | ***p*** | **z** | **Prob > \|z\|** |
| --- | --- | --- | --- |
| 1.Clarity of the SDM | 0.035 | 2.091 | 0.0365 |
| 2.Involve the patient | 0.005 | 2.729 | 0.0063 |
| 3.Different options presented | 0.00015 | 2.966 | 0.0030 |
| 4.Presented advantages/disadvantages | 0.008 | 2.445 | 0.0145 |
| 5.Assisted patient comprehension | 0.010 | 2.855 | 0.0043 |
| 6.Facilitate discussion on preferences | 0.007 | 2.714 | 0.0066 |
| 7.Evaluated together the options | 0.363 | 1.070 | 0.2844 |
| 8.Co-selected. treatment | 0.69 | 0.361 | 0.7180 |
| 9.Reach an agreement | 0.42 | 0.730 | 0.4652 |

Table 4s. UTAUT

|  | ***p*** | **z** | **Prob > \|z\|** |
| --- | --- | --- | --- |
|  |  |  |  |
| Productivity/Perform expectancy | 0,001 | 3.460 | 0.0005 |
| Effort expectancy | 0,001 | 3.722 | 0.0002 |
| Attitude towards using technology | 0,000 | 2.406 | 0.0161 |
| Social influence | 0,001 | 2.874 | 0.0040 |
| Self-efficacy | 0,255 | 1.048 | 0.2949 |
| Facilitating conditions | 0,577 | -0.454 | 0.6496 |
| Anxiety | 0,002 | -2.474 | 0.0134 |
| Behavioral intention |  | -0.165 | 0.8939 |

Table 5s. Physicians’ results for the SUS

|  | ***p*** | **z** | **Prob > \|z\|** |
| --- | --- | --- | --- |
| Logical | 0.03 | 2.268 | 0.0233 |
| Efficient | 0.04 | 2.035 | 0.0419 |
| Effective | 0.016 | 2.514 | 0.0119 |
| SDM use | 0.0008 | 2.817 | 0.0048 |
| SDM valuable | 0.024 | 2.403 | 0.0163 |
| Easy to use | 0.036 | 2.326 | 0.0200 |
| Learn from the experience | 0.064 | 1.781 | 0.0750 |

Table 6s. NASA task load index

|  | ***p*** | **z** | **Prob > \|z\|** |
| --- | --- | --- | --- |
| Mental demand | 0.58 | -0.566 | 0.5711 |
| Easy/demanding | 0.08 | -2.550 | 0.0108 |
| Temporal demand | 0.28 | -0.905 | 0.3656 |
| Effort | 0.3 | -0.575 | 0.5655 |
| Performance | 0.45 | -0.502 | 0.6157 |

Table 7s. *Behavioral intention scale*

|  | ***p*** | **z** | **Prob > \|z\|** |
| --- | --- | --- | --- |
| Conviction to SDM | 0.02 | 2.227 | 0.0259 |
| Control over the SDM level | 0.21 | 1.264 | 0.2063 |
| No time extension | 0.03 | 2.008 | 0.0447 |
| Knowledge importance | 0.20 | 1.369 | 0.1709 |
| Communication skills | 0.17 | 1.483 | 0.1380 |
| Patient motivation | 0.44 | 0.672 | 0.5017 |
| Patient understanding | 0.23 | 1.030 | 0.3032 |

Table 8s. Patients results for the SUS

|  | ***p*** | **z** | **Prob > \|z\|** |
| --- | --- | --- | --- |
| Logical | 0.65 | -0.471 | 0.6374 |
| "Efficient" | 0.77 | 0.316 | 0.7518 |
| "Helpful" | 0.57 | 0.428 | 0.6685 |
| "SDM valuable" | 0.26 | -1.173 | 0.2410 |
| "Easy to use" | 0.83 | 0.000 | 1.0000 |
| "Enjoyed" | 0.55 | -0.638 | 0.5233 |
| "Learn from the experience" | 0.67 | 0.123 | 0.9020 |

Table 9s. Patients Decisional Conflict Scale

|  | ***p*** | **z** | **Prob > \|z\|** |
| --- | --- | --- | --- |
| Decision is hard for me to make | 0.83 | -0.334 | 0.7384 |
| Unsure what to do in this decision | 1.00 | 0.000 | 1.0000 |
| Clear what choice is best for me | 0.57 | -0.424 | 0.6716 |
| I am aware of the choices I have to protect myself from drug interactions | 0.76 | -0.178 | 0.8586 |
| I know the benefits of avoiding drug interactions | 1 | -0.360 | 0.7186 |
| I know the potential for harm for drug interactions | 0.69 | 0.267 | 0.7893 |
| I need more advice and information about my choices | 0.65 | 0.419 | 0.6752 |
| It’s hard for me to decide if the benefits are more important to me than the risks, or opposite | 1 | 0.000 | 1.0000 |
| I feel I have made an informed choice | 0.72 | -0.433 | 0.6654 |
| My decisions show what is most important for me | 0.72 | 0.173 | 0.8626 |
| I expect to stick with my decision | 0.53 | -0.677 | 0.4984 |

Table 10s: Participants’ quotes during the interview after the virtual encounters.

| DDInteract | | Usual Care | |
| --- | --- | --- | --- |
| Physicians | Patients | Physicians | Patients |
| Advantage flipping back and forward with the medications, cardiovascular risk | The tool help to think the DDI medication, I didn’t have any of the information that what provided" | Most of the time I seem to be able to find what I’m looking for and I like Lexi COMP because it also kind of gives you a percentage breakdown of side effects of the drugs, not so much that I recall on the interactions and what the increases are like | I don't want to accept any increase in risk (bleed) |
| The format is great | I don't see any issue with credibility | The warfarin and NSAIDs interaction is well known and established, but I would double check for DOACs | I didn’t know a web DDI checker |
| Like using it, how dynamic it is, how instantaneously adjust the effects of the different attributes | I take other medications as well, if you can combine everything you get the whole picture of what result of different drugs | To me if I have if I tell a patient to monitor closely that there's a sort of assumption that the patient will just take care of it and that they don't necessarily have to come see me or talk about things. | Webs are credible, I  would prefer more visually, less text |
| This is this is perfect, for what you're looking for, which is you're trying to figure out if the patient understands their risk of GI bleed | I learn better by what I see. It's helpful to know what I'm dealing with (dollar signs) I can make an informed decision | Lexi COMP is well enough known that that's probably my go to | I’ve never even knew that there was a drug interaction checker on web md I’ve always just kind of gone in and use it for hey these are my symptoms |
| A question about clarifying the units for lack of a better term I mean it's just like patient year | I feel satisfied with the information you've given me.  I don't feel like I need to render the Internet and Google everything you said | I find the Internet so worthless for medical information to patients | I wouldn't take it reading that yeah I wouldn't take it |
| It's only applicable for GI bleed and that's the disadvantage… , but the idea is excellent. | I learned better by what I see. | I don't need to tell you that the number of drugs and the drummer of drug potential interactions is incredible and they just throw everything at you, and you have no idea to prioritize what's important or not important. | I think a lot of people use the Internet, and I would just stick with My doctor because I’m sure it's the truth.  As just a normal person I don't have medical training, so I would ask my doctor. |
| …and I trust that your tool is based on evidence-based medicine | I have the options of what the price was… You know it's helpful to know | I would be more likely to use this if the patient asked me about a drug drug interaction that I wasn't familiar with | I mean it’d like when it gives you what the interaction is um you know, but it would be kind of Nice and maybe does it tell you what the alternatives are. |
| I felt very confident in the recommendation from the standpoint of risk. | I thought it was very helpful |  | This is just too much I don't even want it, you know I want something more simplistic. |
| Yeah I didn't have any issues with credibility the tool | I thought it was it was much more thorough than the information that I’ve gotten. |  | It was beyond the scope of anything I could understand |
| I liked how dynamic, it was. | Extremely helpful |  |  |
| I think I probably spent a little more time talking about drug interactions and then I would have without the tool, but I think it helped to come to a better decision than I wouldn't without it |  |  |  |
| Overall, this is, this is a good tool, I think this is really useful cool. |  |  |  |
